# An occupational perspective of the lived experience of individuals with Alopecia Areata: A scoping review

**DOI:** 10.1177/03080226251368232

**Published:** 2025-09-11

**Authors:** Lexi Lindley, Daniel Cezar da Cruz, Angela Murphy

**Affiliations:** School of Health, Leeds Beckett University, Leeds, UK

**Keywords:** Alopecia areata, hair loss, occupational participation, occupational engagement, daily life

## Abstract

Alopecia areata (AA) is a chronic condition that causes unpredictable hair loss, often leading to significant psychological distress. While the social and medical implications of AA have been explored, the occupational impact has not been considered. This scoping review aimed to explore the lived experiences of individuals with AA from an occupational perspective. A five-stage methodological framework was followed, with a search conducted across three databases (2003–2023), identifying key terms related to AA and its effects on both children, adults, and older adults. Eleven qualitative studies were selected from an initial 265 results, including 989 participants aged 10–79, mainly from North America and the United Kingdom. Data were analysed thematically, identifying three key themes: (1) Navigating AA through occupations across the lifespan, (2) Occupational Engagement in Self-Care is not Always Pleasurable, and (3) Connecting the meaning of hair and its loss with daily occupations. The review concluded that AA affects various aspects of life, including work, study, self-care, and physical activities. While the condition can be distressing, it sometimes leads to the development of new, meaningful occupations. These findings highlight the potential role of occupational therapists in supporting individuals with AA.

## Introduction

Hair styling, removal and grooming have been perceived with great importance, reflecting social, cultural, spiritual, political and religious beliefs across human existence. Sherrow (2023) notes that historically, various cultures have placed a substantial significance on hair and the removal of hair. The occupation of arranging hair can symbolise individuality, beauty and health, providing psychological value and, potentially, acceptance. A lock of hair has also been considered a memento, providing a connection among loved ones or representing specific periods of life. The removal of hair has been used in wartime as a form of punishment and oppression, and in periods of enslavement to remove individual identity and cause humiliation. Throughout history to the present day, hair continues to have a complex nature, due to its diverse representation and symbolisms (Sherrow, 2023). It is, therefore, not surprising that the loss of hair due to a medical condition is acknowledged in the healthcare sector as a potential cause of distress (National Health Service, 2024).

Alopecia areata (AA), an incurable inflammatory disease, directly compromises hair follicles, resulting in unpredictable hair loss, and fluctuating in severity. Initially, the clinical presentation of AA is commonly associated with circular patches of hair loss on the scalp. However, the disease may progress to alopecia totalis (AT), where the complete loss of scalp hair may occur. AA may advance to alopecia universalis (AU), where all body hair is lost entirely. On-going episodes of recovery and relapse may be experienced, and medical treatment is not guaranteed to be effective (Messenger, 2021). The aetiology of the condition remains unknown (British Association of Dermatologists, 2024), despite AA being estimated to affect 2% of the world’s population (Lee et al., 2020), with a 0.58% prevalence for adults in the United Kingdom (UK; Harries et al., 2022).

Physicians acknowledge the psychological implications of AA (National Institute for Health and Care Excellence, 2023), especially considering this population have a 30-38% increased risk of developing new-onset mental health conditions following an AA diagnosis (Macbeth et al., 2022). Despite this, AA has been considered a cosmetic disease (Barkauskaite and Serapinas, 2020). Data obtained between August 2015 and March 2016 found that six National Health Service (NHS) Trusts in England referred to alopecia as a cosmetic issue when justifying the absence of funding for wig provisions (Johnson and Montgomery, 2017).

## An occupational perspective of individuals with AA

An occupational perspective has been conceptualised as understanding humans as occupational beings (Njelesani et al., 2014). This may include, but is not limited to, exploring how an individual is occupationally engaged. The original term ‘engagement in occupation’ describes the subjective experience and the response to being occupied, beyond an observed performance (Yerxa, 1980). Therefore, occupational engagement can be experienced, emphasising the importance of personal involvement with an occupation (Cruz et al., 2023; Davis et al., 2024; Polatajko et al., 2007a).For example, despite not participating in or ‘doing’ valued occupations such as dancing, swimming, and cycling, individuals with AA can still connect or be involved with these occupations because they have emotional value to them, suggesting they may have unmet occupational needs as they are not performing or participating in the occupations.

An extensive review of the concept of participation in the rehabilitation context clarifies that engagement could be associated with participation. Cogan and Carlson (2018) defined *participation* ‘*as being composed of three essential dimensions: performance, subjective experience, and interpersonal connection*’ (p. 2695). They stated that participation involves opportunities and choices. These two elements are influenced by conditions that can be external or internal to individuals. We can argue that individuals with AA might experience changes in their participation since opportunities to engage (subjective experience) are affected by AA, such as fear at work, emotional responses when wearing hair prostheses and relationships with partners, to mention a few examples.

Occupational participation is the ability to perform an occupation within a meaningful context alongside significant relationships. It is in alignment with one’s values, needs and desires (Egan and Restall, 2022). Therefore, we can assume that enabling occupational participation and engagement may enhance health and well-being (Royal College of Occupational Therapists, 2024). As a consequence of occupational engagement and participation, occupational identity is expressed and created (Christiansen 1999; Christiansen and Haertl, 2024). Across the lifespan, experiences formed from the history of occupational participation become customary to contribute to building one’s occupational identity (Kielhofner, 2008; Taylor et al., 2024). Haircare is considered a predominant part of activities of daily living (ADL), potentially influencing occupational participation within social and community contexts (American Occupational Therapy Association, 2022). This implies speculation that the absence or change of haircare routine may negatively impact occupational participation and the engagement experience.

From an occupational perspective, it appears that AA also has implications for occupational participation in work and leisure. As such, according to Macbeth et al. (2022), an AA diagnosis increases the probability of unemployment by 82%. There is a 56% higher incidence of medically approved absences from work. Despite the study failing to obtain data on the duration or indication of absenteeism, the findings propose that AA has had a prolonged, harmful consequence on productive occupations. Furthermore, AA-related costs have contributed to the accumulation of debt and the forgoing of leisure occupations (Zucchelli et al., 2022).

Prendke et al. (2023) found that the effects of AA may be felt as young as the age of five and have been associated with a reduced quality of life and, in some instances, bullying, influencing school attendance and social occupations. The reduced school attendance possibly indicates that occupational patterns may temporarily be altered for both children and their families, which can be interpreted as a form of occupational disruption (Whiteford, 2000). Likewise, when one is no longer able to do what is required of them or ceases to have the ability to fulfil occupational opportunities, this can diminish or limit occupational potential (Wicks, 2005).

While the medical profession has investigated AA as early as the 18th century (Messenger, 2021), acknowledgement of AA seems to be missing in the Occupational Science or occupational therapy community. In addition, no existing Scoping Review (ScR) is available on the lived experience of the population with AA. This highlights the gap in the literature, justifying the need for our study since their occupational perspective is not fully understood in current research. Therefore, we aim to explore the lived experiences of individuals with AA from an occupational perspective using existing interdisciplinary literature.

## Methodology

A ScR systematically identifies concepts and knowledge gaps on a selected topic (Tricco et al., 2018). This methodology is the most suitable since no evidence has explored AA from an occupational perspective. Hence, a ScR was employed to aid the purpose of this research effectively. A protocol in the format of a research project was formulated by the LL (first author) and supervised by DC (second author). The final manuscript and analysis were reviewed and amended by AM (third author). There is no registration for the protocol of this ScR; however, to address transparency, our research followed the PRISMA Extension for Scoping Reviews, allowing readers enough information to scrutinise and replicate our study (Tricco et al., 2018). Our ScR utilised the five essential stages of the methodological framework proposed by Arksey and O’Malley (2005):

### Stage 1: Identifying the research question

To support the formulation of this ScR research question, the Population, Concept and Context mnemonics were utilised, as advised by Peters et al. (2024). Participants included all genders, from all ages, with AA. Concept comprised all documents containing participants discourse, including all qualitative and mixed-methods research designs if they presented participants’ discourse. To keep a broad focus, the Context included studies from any geographical area if they addressed the other described criterion. The question proposed for this study was: What is the lived experience of individuals with AA from an occupational perspective?

### Stage 2: Identifying relevant studies

Following a consultation with an academic librarian from Leeds Beckett University, Boolean operators were incorporated into keywords and the following search terms: ‘Alopecia Areata’ AND ‘Alopecia’ AND (qualitative OR interview* OR survey* OR ‘lived experience’ OR perceptions*).

Three electronic databases were searched, Discover Database, EBSCOhost and PubMed between the 9th October 2023 and the 28th January 2024. Despite recommendations to be inclusive towards all languages during the search strategy (Peters et al., 2024), the researchers only included journals published in English. A 20-year search limit was defined (2003–2023) to correspond with the establishment of Alopecia UK in 2004 (Alopecia UK, 2024). We only considered open-access publications. As recommended, we screened the reference lists of the materials selected for this ScR to increase the scope of our study (Arksey and O’Malley, 2005; The Joanna Briggs Institute, 2015).

ScRs are notorious for breadth of coverage, including grey literature (Arksey and O’Malley, 2005; Peters et al., 2024). LL manually searched the websites of the World Health Organization (WHO), NHS, Alopecia UK, National Alopecia Areata Foundation, and the European Hair Research Society (EHRS). Nevertheless, no relevant studies were identified regarding the lived experience of this population from these sites. A report was included as it presented participants lived experiences (Food and Drug Administration, 2018).

### Stage 3: Study selection

All results obtained from the electronic databases were initially screened by their title and abstracts. The inclusion criteria were (1) published in English; (2) published between 2003 and 2023; (3) full text; (4) free access. Studies were excluded if they (1) included any other medical condition associated with hair loss, chemotherapy-induced alopecia and scarring alopecia; (2) were from the perspective of a clinician; (3) included another dermatological condition; (4) explored medical implications or treatment; (5) no qualitative data; (6) no abstract available; (7) opinion pieces and letters. All ages were eligible for inclusion, considering that AA diagnosis can occur at any age of life (Lintzeri et al., 2022).

**Figure 1.**
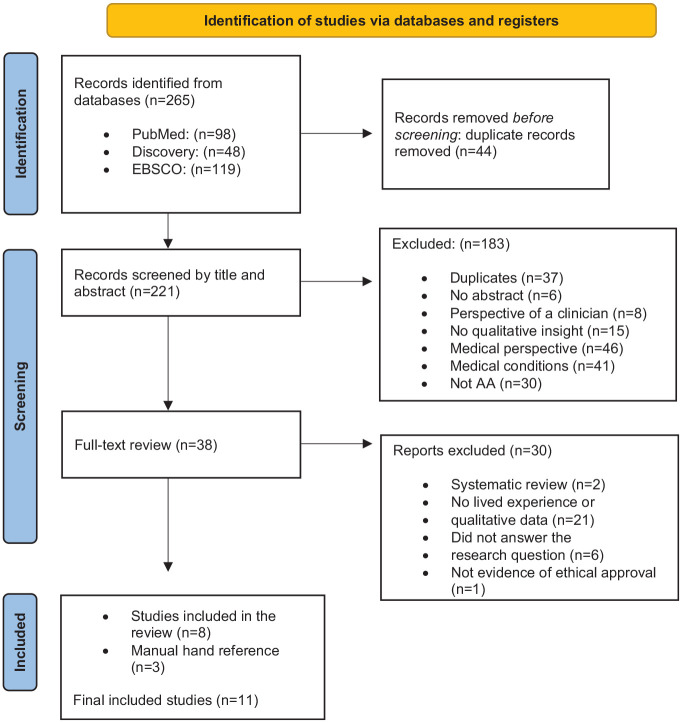
Prisma flow diagram. *Source*. Diagram from: Page et al. (2021).

The search identified 265 studies across three separate databases; 44 were automatically removed due to duplication, and the remaining 221 were screened by their title and abstracts. The remaining 36 underwent a full-text review, and after discussions with DC, systematic reviews were excluded to reduce the risk of duplicated studies. The final selection of studies resulted in a total of 8, and a manual search in their reference lists increased the final selection to 11. The process of screening and selecting studies is documented in Figure 1. As recommended by Tricco et al. (2018), Figure 1 is presented in the results section of this article.

### Stage Four: Charting the Data

Data were extracted from the remaining studies and charted as advised by Arksey and O’Malley (2005) and Peters et al. (2024). For transparency, Tricco et al. (2018) recommend describing whether data charting was performed independently or in duplicate. LL and DC met to discuss what information would be relevant for data extraction. A word/windows form was created specifically for this study. We included the following information: country, year of publication, journal name, and authors. The diagnosis was included since different types of alopecia exist. We extracted information about the number of participants, gender, age, sample specification (children OR adolescents OR adults OR Older-adults OR a mix of them), study design, conclusion and a summary of key findings. Based on Levac et al. (2010), we extracted information for thematic analysis that will be described below.

### Stage 5: Collating, summarising and reporting the results

The Reflexive Thematic Analysis’s six phases (Braun and Clarke, 2022) guided our data analysis. Table 1 summarises the six phases:

**Table 1.** Reflexive thematic analysis phases.

Phases	Description
Phase 1 – Familiarisation	LL initially generated a 33-page Word/Windows file, collating data from the 11 papers. The file was emailed to DC and discussed verbally during face-to-face supervision. Both authors read all articles.
Phase 2 – Coding the data	This phase was initially worked on by LL and revised by DC. Codes were both semantic and latent.
Phase 3 – Generating initial themes	Codes were used to develop themes (Byrne, 2022; Braun and Clarke, 2022). LL created the themes, which were then reviewed and revised by DC and AM. The quotations from participants generated themes from an occupational perspective (deductive analysis). The process was documented in a chart and printed.
Phase 4 – Reviewing and developing themes	LL, DC and AM reviewed each theme, focusing on terminology and content, and ensured that participants’ quotations were used to support the creation of each theme. In theme 1, subthemes were created to illustrate the patterns of meaning across the lifespan.
Phase 5 – Refining, defining and naming themes	Theme 1 was refined, and instead of being named ‘Occupational loss and disengagement’, the authors found the theme did not encompass all the meaning from the analysis; therefore, it was changed to ‘Navigating AA through occupations across the lifespan’ to create a narrative about a variety of occupations, including losses and engaging in new occupations.
Phase 6 – Producing the report	All authors agreed on the final themes after several discussions via MS Teams and face-to-face meetings. We also reduced the number of quotations from participants. Themes were organised from general (occupations across the lifespan) to specific (e.g., self-care and the meaning of hair loss) to create a narrative of their lived experiences.

The researchers were reflexive throughout regarding their positionalities DC and AM as occupational therapists applying an occupational perspective, LL as a person with AA and AM as one of the research team who had provided occupational therapy for people with AA via a fatigue service.

### Bias and rigour

As recommended by ScR guidelines, an experienced academic librarian was consulted to define the best databases and keywords for the study’s purpose. All supervision sessions held have written records to ensure transparency regarding subjective experiences (Suri, 2020). For example, LL and DC discussed their perceptions of AA and how they differ due to different lived experiences and lenses to understand the phenomenon. Considering the principal researcher has personal experience with AA, reflexive discussions and notes were conducted, as advised by Braun and Clarke (2022). To ensure replication and trustworthiness, the PRISMA flow diagram documented the process (Page et al., 2021). As suggested by ScR guidelines, results also present figures and tables to describe how the findings relate to the research objective (Arksey and O’Malley, 2005; The Joanna Briggs Institute, 2015).

### Ethics

Our research received ethical approval from the university’s local research ethics coordinator at Leeds Beckett University. Although ethical approval is not mandatory for literature reviews, ethical concerns still need to be addressed. Murray et al. (2024) stated that it is essential to check if the primary studies had evidence of ethical approval, and when not, the studies should be excluded from the analysis. Following this reasoning, we checked all studies considered for this review regarding ethical approval.

Ten of the original 12 articles reported ethical approval (Aldhouse et al., 2020; Barkauskaite and Serapinas, 2020; Davey et al., 2019; Mesinkovska et al., 2020; Rafique and Hunt, 2015; Rajoo et al., 2020; Welsh and Guy, 2009; Winnette et al., 2021; Wolf and Baker, 2019; Zucchelli et al., 2022). One study was excluded since no ethical procedures were mentioned in any section of the article. Finally, one of the documents wasn’t research published in a journal but a drug report (Food and Drug Report, 2018).

### Analysis

Figure 1 presents the Prisma Flow Diagram (Page et al., 2021), utilised to document the process of selecting sources of evidence for the ScR.

Table 2 presents the studies (*n* = 11) according to the data charting characteristics. A sample specification according to age was included in the data extraction to recognise the implications of AA across the lifespan.

**Table 2.** Data charting characteristics (*n* = 11).

Year	Authors	Country	Participants	Sample specification*	Type of alopecia**	Study design	Key findings	Conclusion
2009	Welsh and Guy	UK	12 (7 women and 5 men)	Adults (between 30/31 and 59 years) (highlighted as two ages reported)	Formal Diagnosis of AA, AU	Qualitative Study: Interpretive Phenomenological Analysis	Psychosocial challenges are evident due to AA/AU. For women, hair loss may affect their perception of femininity.	The response to AA was to conceal symptoms of hair loss. Coping strategies also involved acceptance and optimism.
2015	Rafique and Hunt	Pakistan	8 (3 males and 5 females)	Adolescents (16–19 years)	Formal Diagnoses of AA	Interpretative phenomenological analysis. (interviews)	AA can lead to a loss of confidence. For women, self-esteem was also impacted. Multiple coping behaviours were recognised, including religion.	AA can have a psychosocial impact and raise concerns about the future and physical appearance.
2018	U.S Food & Drug Administration	USA	Approximately 300 (100 in-person/200 online)	Mixed: *Unable to confirm.* *Younger than 6 – older than 50 years*	50 identify as having AA or patient representatives. AA/AU	Public Meeting on patient-focused drug development for alopecia areata	The daily impact of AA can influence activities in leisure, school, sports, relationships, and work, as well as future aspirations and identity.	AA has a negative effect on emotional well-being and mental health and can lead to social isolation and stigma.
2019	Davey et al.	UK	95 (84 women and 11 men)	Mixed: Adults & Older Adults (18–79 years)	Identified as having AU, AT, AA. 6 of 95 responded ‘no’ to a diagnosis of AA (data presented separately)	Qualitative data (online questionnaire)	Daily activities became challenging or were skipped. Hiding hair loss served as both a support and an obstacle in daily life. AA also had adverse effects on mental health.	Psychological distress is linked to the cultural context and significance of hair and may lead to restrictions on their life.
2019	Wolf and Baker	USA	237	Mixed: children and adolescents. (10–19 years)	AA	A mixed-methods research design. (Survey data with interview transcripts and field notes)	AA can evoke fearful feelings about school and preconceptions of hair loss. In some cases, acceptance of AA enhanced self-awareness and compassion.	For children and adolescents, AA can exert a psychological impact.
2020	Mesinkovska et al.	USA	216 (177 women and 39 male)	Mixed: Adult and Older Adults (18 and >65)	Diagnosis of AA (self-reported), which includes overall moderate-to-severe AA	Cross-sectional, quantitative-qualitative survey	AA had negative impacts on work and school, including bullying. AA also affected relationships with family, friends, and romantic partners.	AA adversely affects many aspects of daily life and is a psychosocial burden for the individual.
2020	Aldhouse et al.	North America and Canada	45 (58% per cent women and 42% men)	Mixed: Adolescents, Adults and Older Adults. (15–72 years)	AA (diagnosed by a clinician)	Qualitative interview study (two study rounds)	Self-care routines focus on hiding hair loss. AA can also diminish social interactions and affect relationships.	Concealing hair loss is important and, as a result, affects daily life.
2020	Rajoo et al.	Australia	16: Focus group (8) and Interviews (8)	Adults (18 to 65 years)	Formal Diagnoses of AA AA, AT, AU	Grounded Theory, Focus groups, and semi-structured interviews	Wearing wigs and other methods of concealing hair loss can pose barriers to physical activity. This includes environmental factors, such as the weather. Negative psychosocial effects also increase the tendency to avoid such occupations.	AA can influence participation in physical activity; enablers to address this include accepting the condition.
2020	Barkauskaite and Serapinas	Lithuania	6 (four women and two men)	Adults (23–31 years)	AU/AT	Phenomenological approach (unstructured interviews)	Different stages of acceptance and grieving can involve feelings of fear and anxiety. AA can also generate a complex response regarding fluctuating identity.	The psychological impacts of AA are linked to the distressing loss of one’s perceived self and the processes of grieving or acceptance.
2021	Winnette et al.	USA	36 in total Concept elicitation interviews (20 Adults). Cognitive debriefing interviews (9 adults, 7 adolescents)	Mixed: Adults and adolescents	Concept elicitation interviews: self-reported a clinician's diagnosis Cognitive debriefing interviews: dermatologist-confirmed diagnosis of AA Overall: AA, AT, AU	A Qualitative Study: This research was conducted in two stages: concept exploration and content confirmation	AA can trigger negative responses, leading to decreased activity or avoidance.	AA affects daily routines, especially exercise and outdoor pursuits. Low self-esteem and frustration are also frequently experienced.
2022	Zucchelli et al.	UK	18 (all identified male)	Mixed: Adolescents, Adults and Older Adults (17–71 years)	AA/AU Self-reported	Qualitative thematic analysis (semi-structured interviews)	For men, hair loss can impact their perception of masculinity. AA might also lead to avoiding everyday activities, and efforts are made to conceal it (including the use of wigs, hats and shaving).	Societal expectations within the context of AA for males can heighten isolation; however, they can also foster personal growth.

* Children OR Adolescents OR Adults OR Older-adults OR a mixed of them.

** Alopecia Areata (AA), Alopecia Totalis (AT), Alopecia Universalis (AU).

Based on the findings from this ScR, three themes were created: Theme 1: Navigating AA through occupations across the lifespan; Theme 2: Occupational engagement in self-care is not always pleasurable; and Theme 3: connecting the meaning of hair and its loss with daily occupations.

### Theme 1: Navigating AA through occupations across the lifespan

This theme refers to how individuals with AA expressed their lived experiences through navigating their occupations across their lives. Likewise, ten of the eleven studies acknowledged AA had affected different valued and meaningful occupations of daily life, such as shopping, and childcare (Aldhouse et al., 2020; Food and Drug Administration, 2018; Davey et al., 2019; Mesinkovska et al., 2020; Rafique and Hunt, 2015; Rajoo et al., 2020; Welsh and Guy, 2009; Winnette, 2021; Zucchelli et al., 2022).

#### Sub-theme: Disrupting routines and social roles

Mesinkovska et al. (2020) reported a detrimental impact on routines, including basic ADL, for instance, with a participant expressing: ‘It’s a struggle to get out of bed’ (Mesinkovska et al., 2020: S65). Studies identified reduced or occupational disruption in leisure and social roles (Aldhouse et al., 2020; Davey et al., 2019; Rafique and Hunt, 2015; Zucchelli et al., 2022). The physical appearance caused by AA had consequences on non-participation in public spaces, as one participant stated: ‘*Shortly after the hair loss I was asked to be an usher at a good friend’s wedding, I felt I had to turn it down because I wasn’t ready to be seen in public*’ (Welsh and Guy, 2009: 197).

Two studies described how the environment, including weather or temperature, could adversely concern wearing a hair prosthesis, which can restrict individual participation (Davey et al., 2019; Rajoo et al., 2020). As a result, individuals with AA avoided occupations, from sports to going out shopping, due to anxiety about being observed and exposed (Rajoo et al., 2020).

Five studies discussed the negative impact of AA on participation in physical activity (Aldhouse et al., 2020; Davey et al., 2019; Rafique and Hunt, 2015; Rajoo et al., 2020; Winnette et al., 2021). These studies suggest that AA has the potential to have damaging repercussions towards individuals’ health since participants revealed disruptions from a range of meaningful occupations such as swimming, running, cycling and attending the gym (Rajoo et al., 2020). Specific reasons presented by individuals were related to AA, for instance, giving up gymnastics due to ‘wig malfunctions’ (Wolf and Baker, 2019: 295). In one study, it appears that the experience of living with AA increased addictions that worked as a mechanism of escapism; for example, one participant reported AA influenced increased engagement towards the occupation of smoking (Rafique and Hunt, 2015).

#### Sub-theme: Childhood and adolescence occupations

AA seems to have adverse implications in the early stages of human development; for instance, bullying was discussed as a significant consequence of AA in two studies (Davey et al., 2019; Food and Drug Administration, 2018), with participants missing time from school, repeating academic years, discontinuing education, and expressing upsetting experiences of school participation (Mesinkovska et al., 2020). Participants from the study of Davey et al. (2019) reported physical and psychological violence: ‘I *was teased, bullied and beaten up [in school] because I didn’t have hair. I was called so many names – alien, cancer girl, freak etc – I had my bandannas and hats ripped off my head in the yard, I was beaten multiple times*’ (p. 1382) and similar findings in the study of Barkauskaite and Serapinas (2020).

Wolf and Baker (2019) presented in their study with children and adolescents with AA ages ranging from 10 to 19 years that social issues related to fears of bullying and stares from others. Experiences of bullying created unfavourable impacts on school participation and making new relationships, such as friends. Importantly, physical activity was referred to as the most upsetting occupation by adolescents (Winnette et al., 2021). There are concerns regarding their self-esteem/confidence, psychological effects and impact on socialisation and communication, with 81% of participants avoiding people and 74% reporting preferring doing things alone, and females between the age of 10 and 19 years presenting more difficulties in adaptation, socially and psychologically, compared to males (Wolf and Baker, 2019).

#### Sub-theme: Adulthood and work life

Although the use of hair prostheses could reduce the visual appearance of hair loss, occupational participation and relationships in adulthood were still limited. For example, in the study of Aldhouse et al. (2020) a participant shared the impact on their occupational role as a parent due to the use of hair prosthesis: ‘*I felt like I lost so much with my kids growing up at the beginning because I wasn’t able to be there for them. Like, I’ve never been inside the pool with them. I was always afraid of the wind blowing and everyone noticing that it was a wig*’ (p. 8).

In addition, participation in productive occupations such as education and paid work was found to be an unpleasant experience due to AA (Barkauskaite and Seraphina, 2020; Davey et al., 2019; Food and Drug Administration, 2018; Mesinkovska et al., 2020; Rafique and Hunt, 2015). Participants found work stressful and challenging, and referred to feelings such as being ‘thrown away’ by society after several unsuccessful attempts to gain employment (Barkauskaite and Serapinas, 2020: 3). At work, they referred to unsupportive behaviours from colleagues, missing hours due to AA and resignations that resulted in financial difficulties (Mesinkovska et al., 2020).

#### Sub-theme: Discovering new forms of participation

Conversely, five studies documented how AA has either inspired or influenced different or new forms of occupational participation (Davey et al., 2019; Rafique and Hunt, 2015; Welsh and Guy, 2009; Wolf and Baker, 2019; Zucchelli et al., 2022). These new forms are within areas of education, such as wig-making (Davey et al., 2019); occupations associated with AA charities (Davey et al., 2019; Zucchelli et al., 2022); and changes in spirituality (Rafique and Hunt, 2015), for example, one participant expressed how their own experience living with AA helped to build resilience to support others: ‘*I would like my hair to grow back but I’ve met so many nice people and set up my own business because of it. So, to me it’s been a good thing in a bizarre way, I feel as if I’ve helped people*’ (Welsh and Guy, 2009: 199).

From the perspective of children and adolescents, AA has contributed to personal changes that imply who they really are or their discovery of authentic selves reflecting on their social participation: ‘*It basically just let me out of my shell. I am more a social butterfly now than I was’* (Wolf and Baker, 2019: 294).

### Theme 2: Occupational engagement in self-care is not always pleasurable

As hair loss is the dominant clinical feature of AA, it may not be surprising that nine studies discussed aspects associated with the self-care domain (Aldhouse et al., 2020; Barkauskaite and Serapinas, 2020; Davey et al., 2019; Food and Drug Administration, 2018; Mesinkovska et al., 2020; Rafique and Hunt, 2015; Welsh and Guy, 2009; Winnette et al., 2021; Zucchelli et al., 2022). A coping strategy of AA was to conceal hair loss (Aldhoouse et al., 2020; Barkauskaite and Serapinas, 2020; Davey et al., 2019; Food and Drug Administration, 2018; Mesinjovska et al., 2020; Rafique and Hunt, 2015; Welsh and Guy, 2009). Whilst some males referred to shaving hair to conceal and control AA (Zucchelli et al., 2022), others invested economically in order to maintain their work roles, spending money on wigs, fake lashes and brows, feeling it is critical to look professional (Food and Drug Administration, 2018). Others felt preoccupied and self-conscious with regular checks in the mirror (Winnette et al., 2021). All this evidence suggests changes in occupational engagement.

The concealing of hair loss can create a complex emotional response in individuals’ routines. Despite concealment being considered a helpful strategy, this may provoke feelings of shame, inauthenticity and frustration because they always had the same hairstyle (Barkauskaite and Serapinas, 2020; Davey et al., 2019).

Objects associated with self-grooming tasks, such as using a mirror, triggered intense responses (Davey et al., 2019; Food and Drug Administration, 2018; Meskinkovska et al., 2020). In one instance, the use of a mirror was actively avoided, as the individual no longer recognised themselves (Mesinkovska et al., 2020), generating distressing thoughts and feelings (Davey et al., 2019; Food and Drug Administration, 2018). In summary, evidence illustrates the negative impact on identity and the subjective experience during self-care occupations. This was expressed in distressing thoughts and feelings during and regarding self-care, which reflects unpleasant subjective experience during their self-care, including the act of doing, the objects related to it and the purpose.

### Theme 3: Connecting the meaning of hair and its loss with daily occupations

Seven of the eleven studies provided insight towards the meaning of hair and the experiences of hair loss from a personal perspective (Aldhouse et al., 2020; Barkauskaite and Serapinas, 2020; Davey et al., 2019; Food and Drug Administration, 2018; Rafique and Hunt, 2015; Welsh and Guy, 2009; Zucchelli et al., 2022). Hair comprised the physical appearance looked at first glance by others, which demonstrate a sense beyond the individual, but part of societal expectations and values (Aldhouse et al., 2020).

Other studies reported feelings that hair loss represented a form of punishment (Barkauskaite and Serapinas, 2020), and sadly influenced masking attitudes, behaviours and how individuals presented themselves with clothing: ‘*I just felt that I had to overcompensate with my personality for my lack of hair, always trying to be extra funny and going and buying lots of nice clothes’* (Welsh and Guy, 2009: 197).

Although the lived experience suggests how individuals with AA can possibly have their occupational identity masked, other experiences revealed the opposite. One participant disclosed how their lived experience was seen as a possibility for inner growth, self-acceptance beyond what is considered an ideal stereotype and enjoying the process: *‘I’m definitely a better human being for* . . . *looking inwards rather than externally and seeing the good in me as a person rather than just some superficial hair, body, face* . . . *May sound a bit odd but I wouldn’t change the experience at all’* (Zucchelli et al., 2022: 9).

The same positive attitude could be identified in a child’s perception of being in an expert position allowing them to educate others, for example, in the study of Wolf and Baker (2019), a participant expressed: ‘*I like to educate people when they ask me, ‘what is alopecia’ and I would tell them and they are like ‘Oh, okay. ‘It’s a learning experience for them as it is as much for me*’ (p. 295).

Several studies provided insight regarding the association between hair and femininity or masculinity, which consequently, became impacted negatively due to the loss of hair or haircare occupations and social life (Barkauskaite and Serapinas, 2020; Davey et al., 2019; Welsh and Guy, 2009; Zucchelli et al., 2022), with participants feeling ‘less masculine’ (Zucchelli et al., 2022: 8), or making sexual occupations difficult because they didn’t feel sexy (Mesinkovska et al., 2020).

Furthermore, social stigma resulted in participants receiving comments or believing the public perceived their hair loss as a result of other medical conditions, such as cancer (Aldhouse et al., 2020; Rajoo et al., 2020; Wolf and Baker, 2021), resulting in some cases in avoidance of going to unfamiliar public spaces to prevent emotional discomfort (Zucchelli, 2022). On the other hand, social support from family and friends, engagement with social media and occupations that contribute to wellbeing, such as religious occupations and yoga were recognised by these individuals as a strategy to reduce stress levels (Rajoo et al., 2020). Participants in the study of Rajoo et al. (2020) reported the importance of being with groups that are experiencing the same conditions as critical for acceptance, and this included online forums and specific websites. Additionally, a study with adolescents from Pakistan identified coping mechanisms through engagement in occupations such as watching a movie, spending time studying, playing cricket, making new friends and visiting friends more often (Rafique and Hunt, 2015).

## Discussion

This is the first ScR to gain insight into the lived experience of individuals with AA through an occupational perspective. Five studies were conducted in North America, three in the United Kingdom and one study each from the following countries: Australia, Lithuania and Pakistan. Interestingly, no studies from South America were found. The studies involved 989 participants aged between 10 and 79. Overall, our findings indicate that occupational changes occur due to the consequences of AA, ranging from disruptions and deprivations in school to leisure, work, sexual and self-care occupations. Our discussion is oriented by references to critical occupational therapy to highlight issues of occupational injustices. It is guided by the critical reflexivity of authors who, for example, position themselves in terms of familiarity with the topic and choose to discuss the subject using the theory of occupational justice. The discussion emphasises the need for collaborative approaches aiming at social transformation; for example, individuals with AA can benefit from favourable contexts to enable their choices and control over what they need and want to do, as stated by Whiteford and Townsend (2011).

### Occupational disruption and deprivation across the lifespan

A prominent theme across 10 of the 11 studies is the challenges individuals with AA face concerning their meaningful occupations across various aspects of life and throughout their lifespan. We can argue that changes varied notably in volition and occupational patterns, ranging from temporary to absolute (from occupational disruption to occupational deprivation) across the studies. Most notable were the substantial differences between experiencing occupational disruption and deprivation, in which hair prosthesis and environmental factors could contribute to restricting or re-enabling occupation. These findings are consistent with Montgomery et al.’s (2017) cross-sectional UK survey, which found that wearing hair prostheses for individuals with AA increased social participation, confidence and self-esteem. Conversely, it decreased social occupations and had negative effects on daily life, such as wigs becoming displaced or noticeable. This reflects the dynamic relationship between individuals with AA and their environment, including both physical and social aspects. Therefore, occupational deprivation and disruption can affect individuals in different ways, depending on societal context, relationships and available resources.

Social avoidance was sometimes related to fear or stigma associated with hair loss. The restrictions or demands for appearance in relation to societal norms appeared to shape participants’ thoughts, feelings and behaviours. Rafique and Hunt (2015) argue that anxiety related to appearance results from how individuals perceive stigma, influenced by social norms. Thus, anxiety increases when a person faces a “triggering” event, leading them to adopt coping strategies to reduce anxiety, for example, through avoidance and concealment (Rafique and Hunt, 2015).

Furthermore, AA seems to negatively influence not only individuals’ thoughts and feelings but also their participation in meaningful occupations with others, as participants reported disruptions in co-occupations with their children, sexual relationships with partners, difficulties in engaging with friends at school and work and withdrawal from sports and other physical pursuits. These co-occupations could be crucial in supporting individuals with AA, since evidence indicates that relative acceptance can take time but may be achieved if the social environment is supportive (Barkauskaite and Serapinas, 2020).

Although an ideally supportive environment should enable individuals with AA to participate in society, our findings illustrate the opposite, specifically from social groups expressing prejudice and unacceptable behaviours during their occupational participation. In several studies, participants expressed that AA caused hindrance within school and education, specifically noting discrimination and bullying as a consequence of hair loss. These findings are congruent with a systematic review of the quality of life of children and adolescents with AA (Prendke et al., 2023). Their results uncovered the psychosocial implications of AA, regarding experiences of bullying and reduced occupational participation.

Concerning work, Zucchelli et al. (2022) reported that having AA influenced changes towards work behaviour or different career progression among individuals. Comparable findings from a Survey conducted in the United States identified that 62% of respondents reported different life decisions due to AA with disadvantageous outcomes, including education or career (Mesinkovska et al., 2020). If work choices and occupational behaviours are influenced negatively by having AA, these individuals could be at risk of deprivation and not developing their full occupational potential.

### Societal and gender expectations: The significance of hair

Another finding that stands out from the results reported earlier is the subjective and varied meaning of hair, which reflects cultural and societal perceptions associated with hair loss. We can discuss that although meaningful occupations seem to be uniquely experienced by an individual, they are built on occupational participation that is shaped by societal values and influenced by social groups, including how occupational routines can develop and become associated with specific communities of society (Polatajko et al., 2007a). Likewise, our findings provide insight into how hair loss frequently implicates internal and external gender role expectations, such as femininity and masculinity. These expectations look to be consistent with Clarke-Jeffers et al. (2024), whose study of black women’s lived experience of AA revealed how participants felt their hair symbolised both their culture and femininity. Additionally, it is important to recognise that gender and age-related hair loss can be relatively common in males, and therefore perceived by society as normal, which is not the case for females. This suggests that clinical features of AA may be associated with gender and age, affecting the outcomes of studies.

Another similarity between Clarke-Jeffers et al. (2024) and the findings from our study is the recognition of the profound personal value hair held for some participants, to the extent that, when facing the reality of AA, suicidal thoughts could emerge. Unfortunately, this finding is reflected in higher-than-average incident rates of suicide attempts for the AA population (Wang et al., 2023), suggesting a need for mental health preventative approaches focused on the occupational engagement of individuals with AA.

### Self-care and occupational engagement

A significant finding was how AA influenced change towards occupational participation and engagement in self-care. The physical alterations upon appearance from AA often resulted in the participation in self-care becoming primarily associated with concealing the chronic disease at some stage. Therefore, changing the meaning and purpose of occupational participation and engagement, the latter associated with masking, feelings of guilt, and concerns about other people’s opinions and attitudes. In addition, Aldhouse et al. (2020) found that daily routines associated with personal hygiene became embedded in disguising hair loss through hair prostheses, make-up, cosmetic procedures or shaving of the scalp. This may not, however, appear unexpected, considering that physicians in England and Wales are advised to discuss hair concealment as a form of managing AA. This is currently recommended by the National Institute for Health and Care Excellence (2024), which offers evidence-based clinical guidance to support best practices in healthcare across England and Wales.

If we interpret self-care as a form of occupational engagement, we can raise the question of whether occupational engagement is only a positive experience of participation. Our study shows that participants spend time engaging in self-care, masking, or worrying about their appearance. Polatajko and Davis (2021) refer to occupational engagement as a ‘neutral, non-evaluative concept’ (p. 78), while Fisher and Marterella (2019) affirm that occupational engagement is a synonym of participation and that a person’s value in doing something is not necessarily always positive, but it can be negative or neutral, for example, occupations that are compulsory, obligatory, that are duty or that have values related to acceptance and social connection with the doing. The neutrality of occupational engagement is evident in responses to the loss of haircare routines, as it was not unanimously perceived negatively in the findings of our study.

### The need for advocating occupational justice

An occupational perspective reveals that individuals with AA are facing occupational injustices because they have a range of unmet occupational needs. The disruption and deprivation of occupations they avoid participating in, especially those performed outdoors, are in part due to the appearance caused by the nature of AA. However, it is related to structural factors such as societal values and stereotypes as underlying occupational determinants and contextual issues (Stadnyk et al., 2010; Townsend, 2012). In this case, contextual factors refer to living with AA. Consequently, the thoughts and feelings that prevent individuals from participating and engaging in occupations lead them to occupational marginalisation. This is because they feel discriminated against in opportunities to study and work. Occupational imbalance is identified by their struggle to find a job or isolate themselves, while occupational deprivation manifests through the lack of meaningful occupations, for example, leading them to smoke or abandon physical pursuits that could benefit their health and well-being. Furthermore, they experience occupational alienation due to meaninglessness and lack of control over career decisions and life choices when they try to participate in society. All these forms of occupational concepts are outcomes of occupational injustices (Hocking, 2017; Townsend and Wilcock, 2004).

Misunderstandings and stereotypes regarding AA raise the need to advocate for public awareness about what AA really is. Our ScR is one form of advocacy for individuals with AA to facilitate their participation and engagement in society. As stated by Townsend and Wilcock (2004), occupational injustice is experienced when participation is ‘barred, confined, restricted, segregated, prohibited, undeveloped, disrupted, alienated, marginalized, exploited, excluded or otherwise restricted’ (p. 77).

Individuals with AA experienced circumstances that excluded them from participation and positive occupational engagement. Because they reported limited choices and control of their lives, in some cases, including suicidal ideation, actions need to address these issues. Advocacy, community development and social support could be powerful elements to disrupt barriers that limit their possibilities of choice and control of their occupations (Polatajko et al., 2007b).

Our analysis shows that the lived experience of individuals with AA reveals that expected and actual social responses to differences in participants’ physical appearance negatively affect their occupational experiences and choices. The social model of disability could provide a valuable perspective on how socially constructed stigma contributes to the negative experiences faced by these individuals. In line with an occupational approach, we suggest that the model could emphasise the need for actions at the micro, meso and macro levels, serving as a tool to enhance people's lives (Oliver, 2013).

At the micro level, occupational therapists could work collaboratively with individuals with AA to address their unmet occupational needs and identify local community resources to support their participation and engagement. At the meso level, occupational therapists and individuals with AA could engage with organisations to raise awareness of AA, including charities, schools, workplaces and social media, to find partners and create projects promoting social change. At the macro level, international organisations such as the World Federation of Occupational Therapists and the World Health Organization could engage with policies and generate and disseminate evidence about individuals with AA. As in our study, we can all contribute to ensuring these voices are heard and start a process of change. The Participatory Framework of Occupational Justice presents six enablement skills that can work as an occupational therapy process aligning the aforementioned actions in micro, meso and macro levels: (1) Raise consciousness of occupational injustices faced by individuals with AA, (2) Engage collaboratively with partners, (3) Mediate agreement on a plan, (4) Strategise resource finding, (5) Support implementation and continuous evaluation of the plan and 6. Inspire advocacy for sustainability or closure (Whiteford et al., 2021; Whiteford and Townsend, 2011).

### Implications for occupational therapy and occupational science

Our ScR has applied an occupational perspective to the current evidence on the lived experience of the AA population. Due to the physical alterations associated with hair loss, AA creates a complex response in individuals to their daily occupational participation and engagement in multiple areas across the lifespan.

We argue that, from a healthcare system perspective, there are gaps in knowledge regarding the inclusion of occupational therapists in teams to support people with AA. As experts in using occupation to enhance lives in areas that matter to them, all occupational therapists must advocate for the occupational rights of those with unmet occupational needs or facing injustices (World Federation of Occupational Therapists, 2019). Occupational therapists play a pivotal role in this population with a holistic approach; for example, using the framework proposed by Wilcock and Hocking (2015) in the Occupational Perspective of Health, occupational therapists can design 1:1 and group occupation-centred interventions (focused and based) to facilitate individuals’ occupational participation (doing) in desired roles (being) contributing to their interpersonal relationships with others (belonging) and in accomplishing their occupational goals and aspirations in everyday life (becoming).

In Occupational Therapy Education and Practice Placements, students could work in Role Emerging settings, developing innovative projects to engage with this population, such as peer support groups focused on strategies to promote self-care routines. Occupational therapists could collaborate with GPs in screening for mental health issues related to hair loss, altered body image and its impact on occupational deprivation.

Therefore, preventative actions in secondary care can comprise environmental support to enhance occupational participation, for example, by providing educational support (working in schools) and family support at early stages of AA in children.

### What gaps did this scoping review identify?

Our study highlights an area neglected in occupational science and occupational therapy, emphasising the need for research in the discipline and actions to be taken by the profession at micro, meso and macro levels, advocating for occupational justice.

Moreover, most validated measures available in the research do not identify the everyday living needs of the AA population (Mesinkovska et al., 2020). Therefore, an occupational perspective can reveal the unmet occupational needs, a matter of occupational injustice. Future research could explore the barriers and facilitators experienced by individuals with AA, along with their unmet occupational needs and potential strategies to support their occupational participation and engagement. As our study did not cover all types of alopecia, future studies could examine the experiences of other groups, including various demographics such as age, gender, gender identity, race and ethnicity. By addressing this gap, scholars can generate evidence on the challenges individuals with AA have regarding their occupational participation and engagement. This, in turn, could generate an evidence base to support interventions for occupational therapy practitioners.

### Limitations

Our study has several limitations. The criterion of only including publications published in English suggests that some studies may have been overlooked for review. These criteria may also be aligned with most data obtained from Westernised countries, thereby reducing global applicability perspectives. It is worth noting that there is an under-representation of ethnic minority groups in one-third of the studies of our ScR, which could be, in part, related to the language criteria (Aldhouse et al., 2020; Davey et al., 2019; Mesinkovska et al., 2020; Zucchelli et al., 2022). For the reflexive thematic analysis, we followed Braun and Clarke’s (2022) guidance alongside Byrne (2022), who offers an example of its application. This is not a limitation, but we are aware that due to the combined methodologies, we could not ‘situate’ the participants by providing specific demographics as recommended in the RTA Reporting Guidelines (Braun and Clarke, 2024).

## Conclusion

This is the first study focused on the lived experiences of individuals with AA and their occupational needs. Our ScR has demonstrated, through the synthesis of evidence, that AA has an overwhelmingly harmful effect on occupational participation and engagement, regardless of age. It appears to present fluctuating experiences between occupational deprivation and disruption caused by barriers to wearing a hair prosthesis within the context of the physical and cultural environment, with instances of social avoidance and bullying. On some occasions, this deprivation and disruption were found to negatively affect the occupational roles and sense of efficacy of some participants; the latter was specifically noticeable in areas such as self-care, care for others, work, study and sexual and physical activities. We can then speculate that not participating in desired occupations can negatively impact occupational identity and potential, and future studies should investigate these concepts with people with AA. Occupational therapists are uniquely positioned to address these unmet occupational needs as they can identify the impact on occupational participation and engagement, supporting people with AA to develop strategies for improved health and well-being. Our analysis raises further questions about our role within the dermatology sector and multidisciplinary teams in healthcare at large.

**Key findings**

- Individuals with AA experience difficulties in participation and engagement in occupations across the lifespan, including study, work, leisure, sexual and physical activities.
- Discovering new occupations was a positive factor in sustaining occupational participation and engagement of individuals with AA.

**What the study has added**

- The lived experience of individuals with AA is characterised by occupational disruption and deprivation, influenced by body image and social environment factors.
